# A novel 6 Fr inside biliary stent system for perihilar biliary drainage

**DOI:** 10.1055/a-2767-1949

**Published:** 2026-01-30

**Authors:** Hiroshi Kawakami, Naomi Uchiyama, Hiroshi Hatada

**Affiliations:** 1 12952 Division of Gastroenterology and Hepatology, Department of Internal Medicine, Faculty of Medicine, University of Miyazaki, Miyazaki, Japan

**Fig. 1:**
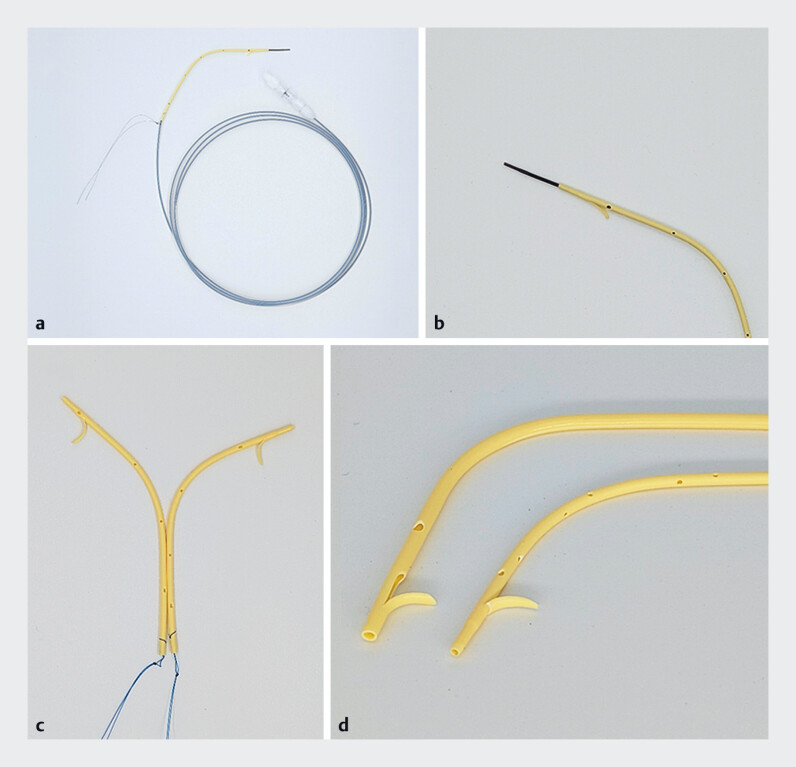
**a** A novel 6 Fr inside biliary stent (9- and 12-cm length) and blue snare, and a 6-Fr pushing catheter system (190 cm in length, SILUX Co., Ltd, Saitama, Japan). **b** The tip of a 3 Fr inner tapered catheter and a 6 Fr inside biliary stent. **c** Novel 6 Fr inside biliary stent placement in a side-by-side manner. **d** Comparison of a 7 Fr inside biliary stent (left) and a 6 Fr inside biliary stent (right).

Endoscopic biliary drainage (BD) is commonly performed in patients with perihilar biliary obstruction; however, the most appropriate method remains controversial. The plastic stent technique involves placing the tip of inside stent within the bile duct. Recently, the clinical outcomes of the inside and conventional plastic stents or the metal stent for the management of BD have been reported to be comparable in patients with preoperable/unresectable cholangiocarcinoma 1 2 3 . However, severe biliary strictures may not allow multiple inside stent placements, and another a replacement is occasionally difficult to advance through the biliary stricture, and involves a risk of migration. To overcome this limitation, we developed a 6 Fr inside biliary plastic stent ( Fig. 1 **a–d** ). Multiple 6 Fr stents can be deployed simultaneously in a side-by-side fashion over the guidewire. Herein, we present patients treated successfully for perihilar BD using this new system.

**Fig. 2:**
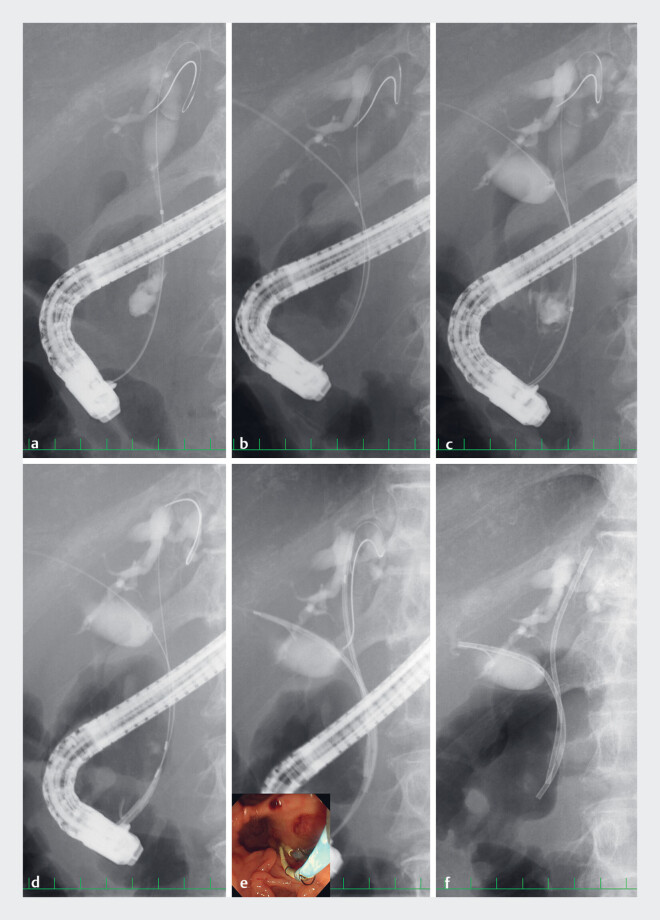
(Case 1) **a** A radiograph showing the left hepatic duct obstruction. **b** A radiograph showing a 0.025-inch guidewire placed in the right hepatic duct. ( **c** ) A radiograph showing the bismuth type IV biliary obstruction. **d** A radiograph showing the novel 6 Fr inside biliary stent inserted in the bile duct. **e** A radiograph showing both stents placed simultaneously in the bilateral hepatic ducts (inset: an endoscopic image). **f** A radiograph showing after extraction inner and pushing catheters.

**Fig. 3:**
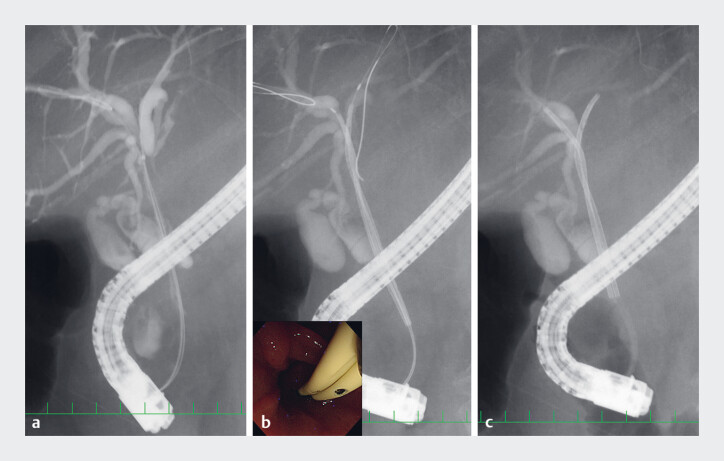
(Case 2) **a** A radiograph showing the bilateral 0.025-inch guidewire placed in the right and left hepatic ducts. **b** A radiograph showing both stents placed simultaneously in the bilateral hepatic ducts (inset: an endoscopic image). **c** A radiograph showing the removal of guidewires and inner catheters.

Case 1: An 89-year-old man with malignant perihilar cholangiocarcinoma was admitted with acute cholangitis caused by dysfunction of a previously placed 7 Fr inside biliary stent in the left hepatic duct. After extracting the occluded biliary stent, endoscopic retrograde cholangiography (ERC) confirmed bismuth type IV perihilar biliary obstruction. The 0.025-inch guidewires were placed in the bilateral hepatic ducts. We successfully deployed 6 Fr inside biliary stents in the bile duct along the guidewire without complications ( Fig. 2 **a–f** , Video 1 ). Case 2: A 78-year-old woman with IgG4-sclerosing cholangitis was admitted to our hospital. ERC confirmed perihilar biliary obstruction. We placed 6-Fr inside biliary stents simultaneously in the bile duct ( Fig. 3 , Video 1 ).

A novel 6 Fr inside biliary stent system for perihilar biliary drainage. The stents can be deployed simultaneously in a side-by-side fashion over the guidewire.Video 1

In conclusion, this novel 6 Fr inside biliary stent system is useful for treating perihilar biliary obstruction. We believe that this system is a new drainage option and the least invasive approach available for the management of BD in patients with benign and malignant perihilar biliary obstructions.

Endoscopy_UCTN_Code_TTT_1AR_2AZ
